# MiR-133a Is Functionally Involved in Doxorubicin-Resistance in Breast Cancer Cells MCF-7 via Its Regulation of the Expression of Uncoupling Protein 2

**DOI:** 10.1371/journal.pone.0129843

**Published:** 2015-06-24

**Authors:** Yuan Yuan, Yu Feng Yao, Sai Nan Hu, Jin Gao, Li-Li Zhang

**Affiliations:** 1 Department of Chemotherapy, Jiangsu Cancer Hospital and Research Institute, Nanjing, Jiangsu, People’s Republic of China; 2 Department of Surgery, Jiangsu Cancer Hospital and Research Institute, Nanjing, Jiangsu, People’s Republic of China; Wayne State University School of Medicine, UNITED STATES

## Abstract

The development of novel targeted therapies holds promise for conquering chemotherapy resistance, which is one of the major hurdles in current breast cancer treatment. Previous studies indicate that mitochondria uncoupling protein 2 (UCP-2) is involved in the development of chemotherapy resistance in colon cancer and lung cancer cells. In the present study we found that lower level of miR133a is accompanied by increased expression of UCP-2 in Doxorubicin-resistant breast cancer cell cline MCF-7/Dox as compared with its parental cell line MCF-7. We postulated that miR133a might play a functional role in the development of Doxorubicin-resistant in breast cancer cells. In this study we showed that: 1) exogenous expression of miR133a in MCF-7/Dox cells can sensitize their reaction to the treatment of Doxorubicin, which is coincided with reduced expression of UCP-2; 2) knockdown of UCP-2 in MCF-7/Dox cells can also sensitize their reaction to the treatment of Doxorubicin; 3) intratumoral delivering of miR133a can restore Doxorubicin treatment response in Doxorubicin-resistant xenografts *in vivo*, which is concomitant with the decreased expression of UCP-2. These findings provided direct evidences that the miR133a/UCP-2 axis might play an essential role in the development of Doxorubicin-resistance in breast cancer cells, suggesting that the miR133a/UCP-2 signaling cohort could be served as a novel therapeutic target for the treatment of chemotherapy resistant in breast cancer.

## Introduction

To develop optimum strategy for overcoming Doxorubicin resistance is one of the major concerns in current breast cancer treatment[1]. Everlasting endeavors have been made in identifying genes related to chemo-resistances in the past decades [2,3]. However, the link between specific genes and Doxorubicin resistance in breast cancer remains unclear.

Uncoupling proteins (UCP) are a subgroup of mitochondrial anion transporters, which consists of three structurally close members, UCP1/2/3[4]. Among these three members, UCP-1 is highly expressed in the mitochondria brown adipose tissue and functions as a thermo-generating protein for thermogenesis[5]. Regarding UCP-2, it was first identified with UCP-3 in 1997 as homologs of UCP-1[6]. Accumulating evidences showed that UCP-2 is expressed ubiquitously and involved in a variety of physiologic processes, including cellular energy expenditures[7], proton leak[8], ATP synthesis[9] and mitochondrial ROS generation[10]. It was reported that increased expression of UCP-2 was found in many cancer cell lines, which might be related to the shifted energy metabolism status of cancer cells[11]. Interestingly, recent findings indicate that UCP-2 might play a functional role in the process of cancer cell biology[12].Its involvement in chemotherapy resistance suggests this mitochondrial uncoupling protein might serve as a potential therapeutic target for cancer treatment [13–15].

MiRNAs are a subgroup of non-coding RNA and characterized as highly conserved its22-nt single-stranded RNAs that suppress the translation of target genes or induce messenger RNA (mRNA) degradation via their binding to the 3’-untranslated region[16]. Increasing evidences demonstrated that miRNAs epigenetically regulate the expression of genes involved in fundamental cellular processes such as cell proliferation, apoptosis, differentiation, and migration, suggesting their potential oncogenic or tumor suppressing roles in cancer development[17]. Recently studies evidenced the reduced expression of miR-133a in a serial of cancers originated from breast[18], lung[19], colon [20] and ovarian[21], which is accompanied by poor prognosis and malignant progression. It was also reported that restoration of miR133a in breast cancer cells could inhibit their cell proliferation and invasion[18], indicating its functional role in breast cancer cell growth.

Based on the findings that miR133a functionally regulates the expression of UCP-2 in muscle cells and macrophages [22,23] and the significances of UCP-2 in chemotherapy resistances of cancer cells, it is postulated that miR133a might play a role in the chemotherapy resistance of breast cancer cells by regulating the expression of UCP-2. In the present study, we aimed to explore the functional significance of miR133a-UCP-2 axis in the Doxorubicin-resistance in breast cancer cells by *in vitro* and *in vivo* cell growth studies. Our results showed that elevated expression of UCP-2 was found in MCF-7-Dox cell line, which is concomitant with reduced expression of miR133a. UCP-2 overexpression in breast cancer cell line MCF-7 promoted their cell proliferation and induced the resistance to Doxorubicin *in vitro* and *in vivo*, which was attenuated by restoration of miR133a. Furthermore, knockdown of UCP-2 in breast cancer cell line MCF-7 decreased their cell proliferation and eradicated its resistance to Doxorubicin *in vitro* and *in vivo*. Moreover, it is also observed that miR133a could also decrease luciferase activity of UCP-2 gene promoter. These findings suggest that miR133a might inhibit Doxorubicin-resistance in breast cancer, which is mediated via its regulation of UCP-2. UCP-2 might be a novel therapeutically target for Doxorubicin-resistant breast cancer therapy.

## Materials and Methods

### Cell culture

Human breast carcinoma cell line, MCF-7, were obtained from the American Type Culture Collection (ATCC, Manassas, VA, USA.). The cells were maintained in RPMI-1640 supplemented with 10% fetal bovine serum (FBS), 100U/ml penicillin and 100μg/ml streptomycin at 37°C in 5% CO_2_ incubation. MCF-7/Dox cells were developed from the parental MCF-7 cells by stepwise selection for resistance with increasing dose of Doxorubicin and maintained in the presence of Doxorubicin (0.37 nmol/L).

### Real-time PCR analysis

Total RNA was extracted from cultured cells using TRIzol reagent (Invitrogen). DNaseI-treated RNA was used for first strand cDNA synthesis using M-MLV reverse transcriptase (Promega) and oligo (dT) _15_ according to the manufacture’s protocols and 1μl cDNA samples were used for conventional PCR amplifications. Real-time quantitative PCR analysis was performed in a real-time PCR system (StepOne, Applied Biosystems) and the expression levels of UCP-2were normalized to GAPDH determined by a SYBR Green-based comparative cycle threshold CT method.Real-time PCR primers were: UCP-2-F: 5’- CTCAGAAAGGTGCCTCCCGA-3’, UCP-2-R: 5’- ATCGCCTCCCCTGTTGATGTGGTCA -3’; GAPDH-F: 5’- TGTGGGCATCAATGGATTTGG -3’, GAPDH-R: 5’- ACACCATGTATTCCGGGTCAAT -3’; miR-133a-F: 5’- ACACTCCAGCTGGGTTGGTCCCCTTCAACC -3’, miR-133a-R: 5’- CTCAACTGGTGTCGTGGAGTCGGCAATTCAGTTGAGACAGCTGG -3’; U6-F: 5’- CTCGCTTCGGCAGCACA -3’, U6-R: 5’- AACGCTTCACGAATTTGCGT -3’. The UCP-2 gene promoter sequences were designed as previously described[22].

### Western-blot analysis

Thirty microgram of cell lysates and tumor tissue lysates were separated on 12% sodium dodecyl sulfate-polyacrylamide gel electrophoresis (SDS-PAGE) gels and then transferred onto nitrocellulose membranes. Specific monoclonal anti-UCP-2 (ab67241) and monoclonal anti-β-actin (ab3280) primary antibodies (Abcam Biotechnology) were used, and HRP conjugated immunoglobulin was used as a secondary antibody (Jackson ImmunoResearch Laboratories). West Pico Chemiluminescent (Pierce) was used as the substrate to visualize protein bands, which were quantified using densitometry image analysis software (Image Master VDS; Pharmacia Biotech).

### *In vitro* cell growth assays

Cell counting. Viable cells were counted as described previously[24]. When thecells were cultured to 70–80% confluence, Doxorubicin was added accordingly. All counts were performed in triplicate wells and repeated in three independent experiments and mean ± SEM of cell number was plotted against culture duration 8 days. For the Doxorubicin treatment assay, cells were seeded according to the doubling time to ensure comparable cell amount.

MTT assay. Cells were seeded at 5×10^3^ cells/well and with or without Doxorubicin. Viable cells were determined by MTT assay as described previously[25].

### RNA interference

For UCP-2 gene knockdown, a set of human UCP-2 shRNA (SH-005114-01-110, Thermo Scientific Open Biosystems) expressing short hairpin RNA (shRNA) targeting to UCP-2 mRNA and control shRNA containing scrambled sequence were used. Lenti-virion were produced in transfected 293FT packaging cells as described previously[26].

### *In vivo* tumor study

Female nude mice (4–6 weeks, 18–20 g) were purchased from the Model Animal Research Center of Nanjing University. MCF-7/Dox cells (5×10^6^, resuspended in 100 μl saline) were injected into the right flank of each mouse subcutaneously. Tumor volumes were determined every five day after inloculation and calculated as described previously [27]. Mice with the volume of xenografts lager than 500 mm^3^ were treated with Doxorubicin(Dox)with the dosage of 4 mg/kg intraperitoneally (*i*.*p*.) combined with intratumoral injection of saline (Dox plus saline group), scramble (Dox plus scramble group) and pre-miR-133a (Dox plus pre-miR-133a group) twice a week for four weeks. Mice were sacrificed and tumors were dissected and processed for real-time PCR and Western blot analysis. All the animal study was carried out in strict accordance with the recommendations in the Guide for the Care and Use of Laboratory Animals of the Jiangsu Cancer Hospital and Research Institute Animal Care and Use Committee. All the protocols were approved by animal care and use committee in Jiangsu Cancer Hospital and Research Institute (approved number 21040608). All the surgeries were performed under sodium pentobarbital anesthesia, and efforts were made anyway to minimize suffering.

### Plasmid Construction and Luciferase Assay

The 3’-untranslated region (UTR) of human UCP-2 gene was amplified by PCR using mouse genomic DNA as a template. The PCR products were inserted into the p-MIR-report plasmid (Ambion). For luciferase reporter assays, 1 μg of firefly luciferase reporter plasmid, 0.5 μg of β-galactosidase expression vector (Ambion), and equal amounts (200 pmol) of pre-miR-133a or scramble shRNA were transfected into cells in 6-well plates. The β-galactosidase vector was used as a transfection control. Cells were assayed using luciferase assay kits (Promega) 24 hours after transfection.

### Statistical analysis

All results were expressed as mean ± SEM from at least three independent experiments. For multiple comparisons each value was compared by one way ANOVA following Dunnett test, Tukey test and Student t-test in GraphPad Instat version 5.0and P values of less than 0.05, 0.01 and 0.001 were considered significant.

## Results

### 1. Decreased expression of miR133a was concomitant with increased expression of UCP-2 in Doxorubicin-resistant breast cancer cell line MCF-7/Dox as compared with its parental cell line MCF-7

**Fig 1:**
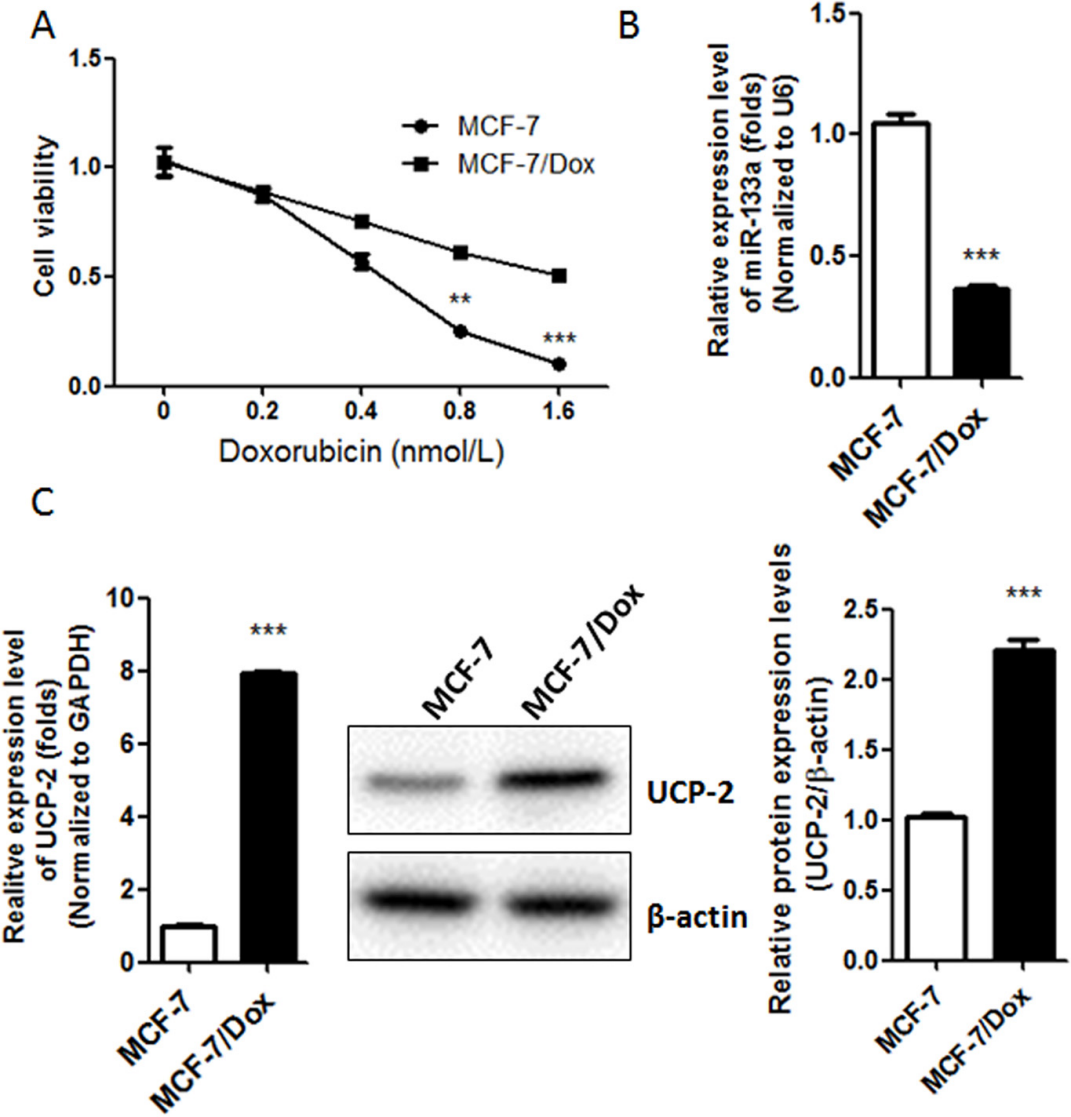
Decreased expression of miR-133a is concomitant with increased expression of UCP-2 in Doxorubicin-resistant breast cancer cell line MCF-7/Dox. (A) The cell viability of Doxorubicin-resistant breast cancer cells MCF-7/Dox and the parental breast cancer cell line MCF-7 after treated with different concentration of Doxorubicin. (B) Real-time PCR analysis of the relative expression of miR-133a normalized to U6 RNA in MCF-7 and MCF-7/Dox. (C) Real-time PCR and western blot analysis of the expression levels of UCP-2 in MCF-7 and MCF-7/Dox cells. Left, the expression levels of UCP-2 mRNA relative to GAPDH were determined by a SYBR Green-based comparative cycle threshold CT method.Right, Antibody against UCP-2 was used in this assay. Protein loading control was done with anti-β-actin.Each bar represents the mean ± SEM. The results shown were repeated in three independent experiments. **, P < 0.01; and***, P < 0.001 significantly different from the respective control group.

The Doxorubicin-resistant cell line MCF-7/Dox was generated by intermittent exposure of sensitive parental MCF-7 cells to 0.37 nM Doxorubicin in RPMI 1640 culture medium for about 1 year. Obvious morphological and cell viability changes were observed after the treatment of doxorubicin (S1 Fig).The Doxorubicin resistant phenotype further was verified by MTT assay (Fig 1A). The IC50 of MCF-7/Dox was 1.49±0.13 nM while that of the Doxorubicin sensitive cell line MCF-7 was 0.68±0.09 nM. The miR-133a and UCP-2 mRNA levels in MCF-7/Dox and MCF-7 were evaluated by quantitative PCR. Decreased expression of miR-133a was observed in MCF-7/Dox as compares with MCF-7 (0.37-flod) (Fig 1B). More interestingly, it was observed that the expression of UCP-2 was increased in MCF-7/Dox at both protein and mRNA levels (Fig 1C), which indicates that miR133a/UCP-2 might be involved in the development of Doxorubicin resistance in breast cancer cell line MCF-7.

### 2. MiR-133a sensitized Doxorubicin response in Doxorubicin-resistant breast cancer cell sub-line MCF-7/Dox via its direct regulation of UCP-2 expression

**Fig 2:**
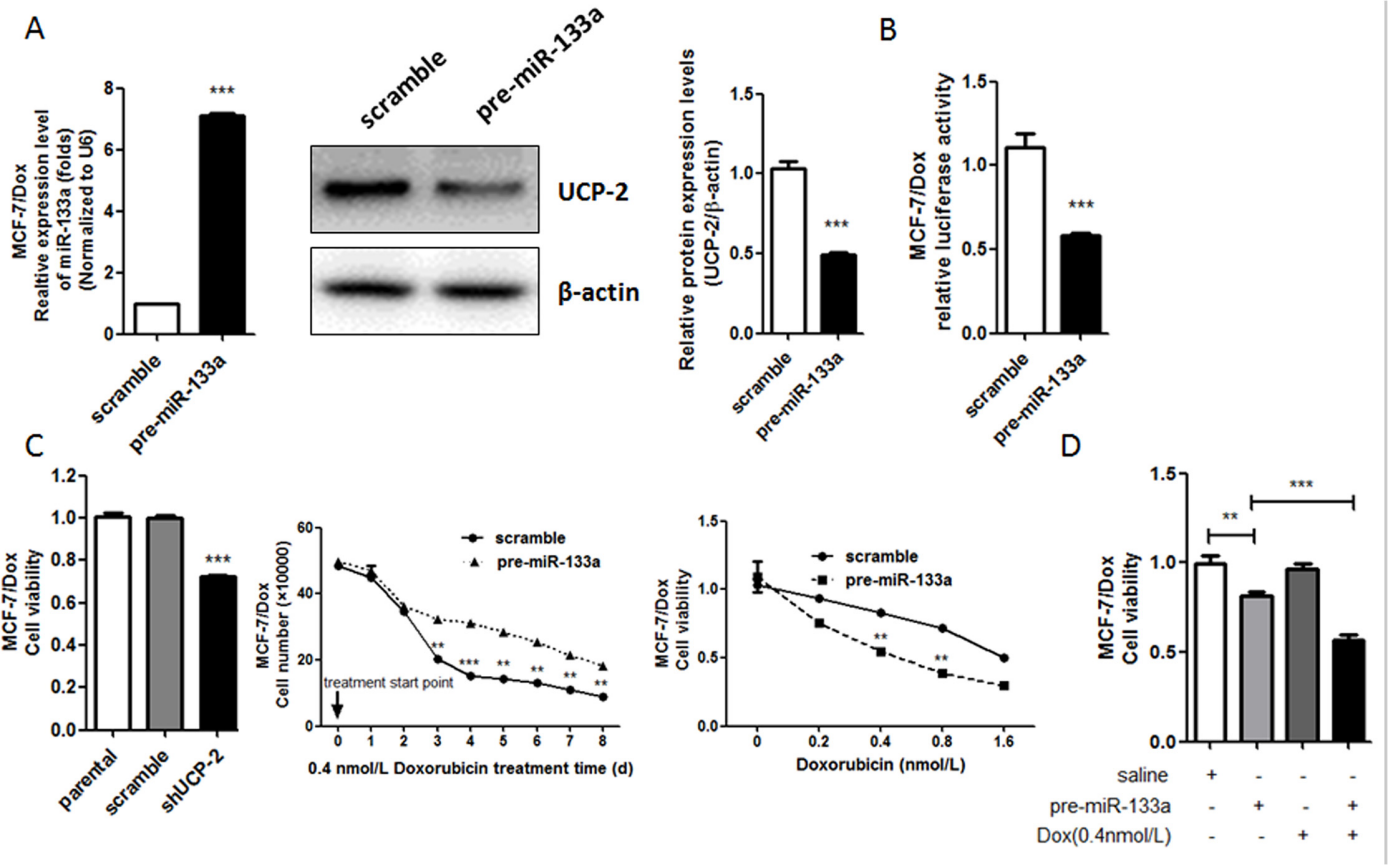
MiR-133a sensitizes Doxorubicin response in Doxorubicin-resistant breast cancer cell sub-line MCF-7/Dox via its directly regulating the expression of UCP-2. (A) MCF-7/Dox cells were transfected with the precursor oligonucleotides (pre-miR-133a and scramble). MiR-133a levels were detected by real-time PCR (Left) and UCP-2 protein levels were determined by western blot analysis of total lysate on 48 h after transfection (Right). (B) Effect of miR-133a expression vector on the luciferase activities of UCP-2 3’-UTR in MCF-7/Dox cells. Firefly luciferase reporters containing UCP-2 3’-UTR were co-transfected into MCF-7/Dox cells with the indicated precursor oligonucleotides. Luciferase activity was determined 24 h after transfection. (C) Cell growth curve plotted with total cell amount of MCF-7/Dox cells transfected with miR133a or scramble sequences by cell counting in connective 8 days treated with 0.4 nM Doxorubicin (Left). Cells were seeded in amount according to their doubling time to ensure comparable amount in the starting day of treatment. Right, cell viability under the treatment of Doxorubicin was shown as folds change of cell viability normalized to that of the cells treated with saline. Each bar represents the mean ± SEM. The results shown were repeated in three independent experiments. (D) MCF-7/Dox cells were treated with pre-miR-133a alone or combined with Doxorubicine. The cell growth inhibition was measured by MTT assay. Each bar represents the mean ± SEM. The results shown were repeated in three independent experiments. **, P < 0.01; and***, P < 0.001 significantly different from the respective control group.

Next, functional role of miR133a in the Doxorubicin resistance in breast cancer cells was explored by gain-of-function study in MCF-7/Dox. It was observed that the expression of UCP-2 was significantly down regulated in miR133a-overexpressed MCF-7/Dox in both protein and mRNA levels as compared with that in the scramble control cell line (Fig 2A). Moreover, the dual luciferase assay showed that transduction of pre-miR-133a in MCF-7/Dox could inhibit gene promoter luciferase activities of UCP-2 3’-UTR (Fig 2B). These findings provide evidences that miR133a could directly regulate the expression of UCP-2.

The proliferation of MCF-7/Dox cells transfected with scramble and pre-miR-133a decreased significantly as indicated by cell counting, while the cytotoxicity of Doxorubicin to MCF-7/Dox was increased significantly as indicated by decreased cell viability (Fig 2C). To exclude the anti-tumor effect of miR-133a on the Doxorubicin resistance role of miR-133a in MCF-7/Dox cells, we measured the cell growth inhibition of miR-133a alone treatment or miR-133a and Doxorubincin combine-treatment in MCF-7/Dox cells by MTT. The results showed that miR-133a and Doxorubicin combine-treatment exhibited a higher cell growth inhibition when compared to the miR-133a alone treatment in MCF-7/Dox cells (Fig 2D).

### 3. Knockdown of UCP-2 in MCF-7/DOX restored its Doxorubicin sensitivity

**Fig 3:**
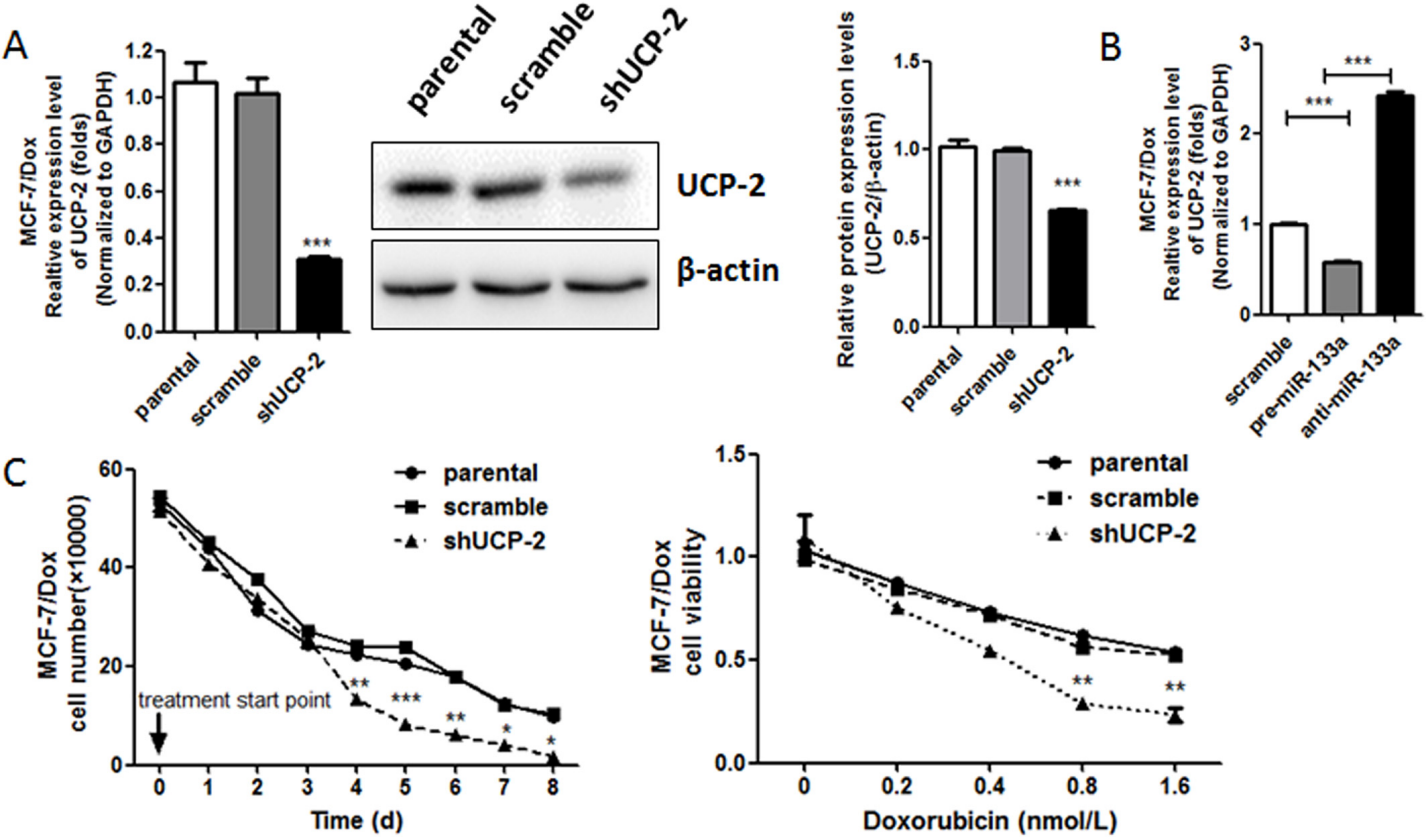
Knockdown of UCP-2 in MCF-7/DOX restores its Doxorubicin sensitivity. (A) The mRNA and protein expression levels of UCP-2in MCF-7/Dox cells infected with lentivirus expressing eithershUCP-2 or scramble shRNA for 72 h were measured by real-time PCR (Left) and western blot analysis (Right) respectively. (B) Cell growth curve plotted with total cell amount of MCF-7/Dox cells infected with lentivirus expressingscramble shRNA and shUCP-2by cell counting in connective 8 days treated with 0.4 nM Doxorubicin (Left).Cells were seeded in amount according to their doubling time to ensure comparable amount in the starting day of treatment. Right, cell viability under the treatment of Doxorubicin was shown as folds change of cell viability normalized to that of the cells treated with saline. Each bar represents the mean ± SEM. The results shown were repeated in three independent experiments. *, P < 0.05; **, P<0.01 and***, P < 0.001 significantly different from the respective control group.

To further validate whether UCP-2 plays a role in the exogenous expression of miR133a-induced sensitivity to Doxorubicin in MCF-7/Dox, the functional significances of UCP-2 gene knockdown were investigated. The results showed that UCP-2 specific shRNA, but not scramble shRNA could significantly reduce the expression of UCP-2 in both mRNA and protein levels (Fig 3A). Interestingly, decreased cell proliferation rate was observed in MCF-7/Dox transduced with UCP-2 shRNA compared to shScramble, which indicated that UCP-2 might mediate the Doxorubicin resistance in breast cancer cells. Moreover, it was also observed that the IC50 of Doxorubicin in MCF-7/Dox transduced with UCP-2 shRNA was significantly lower than that in MCF-7/Dox transduced with shScramble as well as in the parental cells (Fig 3B), which further suggested that UCP-2 plays an essential role in the development of Doxorubicin resistance in breast cancer cells MCF-7.

### 4. MiR133a enhanced the response to Doxorubicin treatment *in vivo* by decreasing the expression of UCP-2

**Fig 4:**
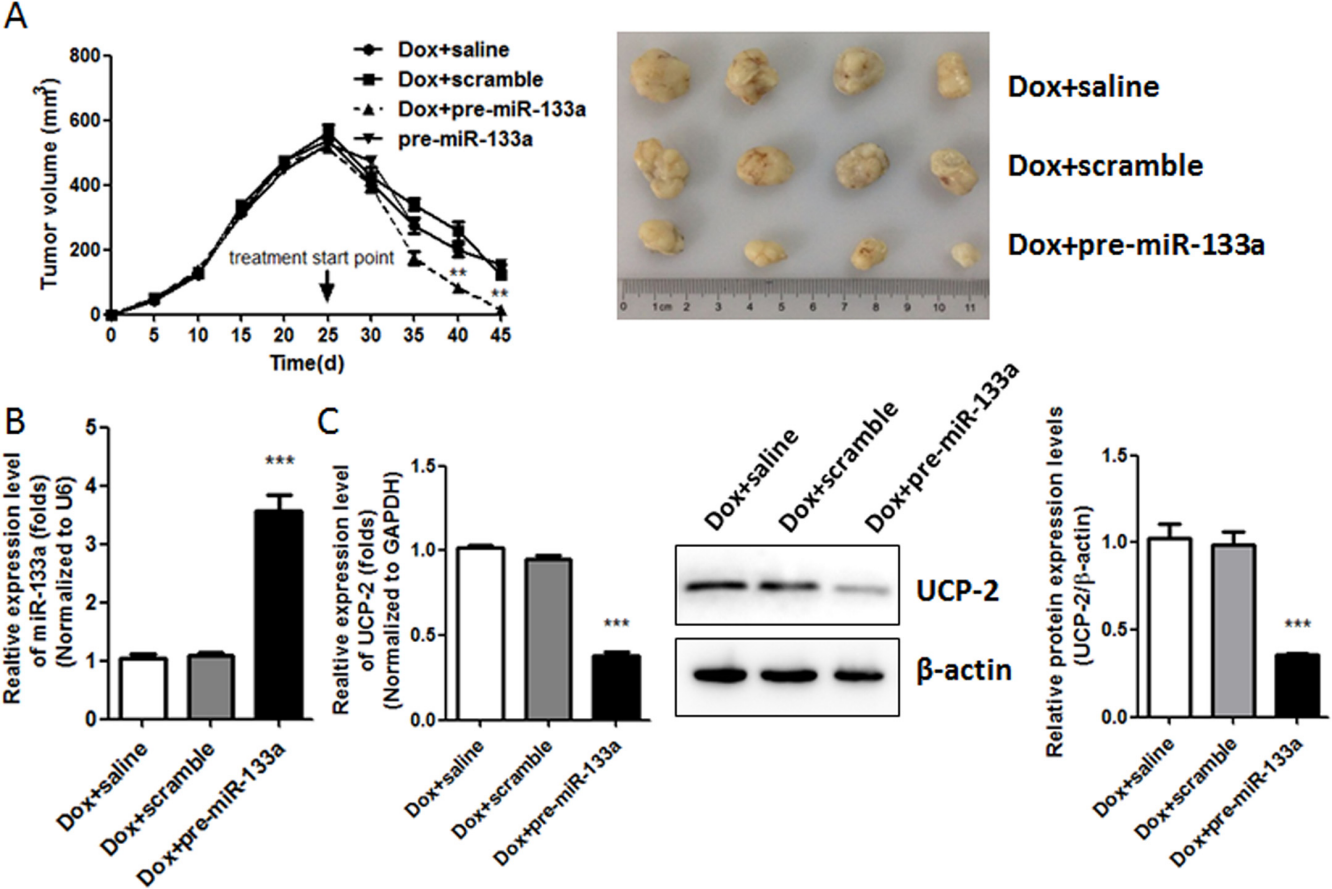
MiR133a helps to increase Doxorubicin treatment response inDoxorubicin-resistant in vivo via its decreasing the expression of UCP-2. (A) Tumor growth curve in the nude mice treated with Doxorubicin intraperitoneally (*i*.*p*.) (4 mg/kg, twice per week) combined with intratumoral injection of pre-miR133a, scramble or saline for four weeks. (B) Expressions levels of miR-133a were detected by real-time PCR analysis in tumor tissues. (C) Expression levels of UCP-2 in tumor tissues were determined by real-time PCR (Left) and Western blot analysis (Right), respectively. Each bar represents the mean ± SEM. All the results shown were repeated in three independent experiments. **, P < 0.01; and***, P < 0.001 significantly different from the respective control group.

Based on the findings that exogenous expression of miR-133a in breast cancer cells attenuated its Doxorubicin resistance *in vitro* possibly by reducing the expression of UCP-2, we further explored the effect of overexpressing miR133a in tumor xenografts *in vivo*. The results showed that pre-miR-133a, but not scramble or saline, could enhance the inhibition effect of Doxorubicin in tumor growth (Fig 4A). The higher expression level of miR133a in tumor xenografts delivered with pre-miR133a as compared with that in tumor xenografts delivered with scramble and saline were validated by real-time PCR, which is concomitant with decreased expression of UCP-2 in mRNA and protein levels (Fig 4B). These *in vivo* findings coincided with what was found *in vitro*, provided direct evidences that the miR133a/UCP-2 axis might be a novel therapeutic target for conquering Doxorubicin resistance in current breast cancer treatment.

## Discussion

Resistance to the treatment of anthracyclines Doxorubicin is one of the major obstacles in current metastatic breast cancer chemotherapy[28]. Increasing evidences indicate that targeting chemotherapy-resistance inducers would be one of the promising strategies to improve the efficacy of treatment[21,29]. Although great endeavors have been made, clinically effective drugs targeting chemotherapy-resistance inducers are still under developed, which may be attributed to the complexity of the development of chemotherapy resistance. To this end, a Doxorubicin-resistant breast cancer cell line, MCF-7/Dox, was established in the present study, which could simplify and better mimic the development of anthracyclines-resistance in breast cancer *in vitro*.

As a metabolic regulator, UCP-2 was found to be involved in cancer progression, which may be mediated by its regulatory role in cancer cell metabolism and correlation to P53 [13]. It was evidenced that overexpression of UCP-2 in colon cancer cells could promote their resistance to topoisomerase I inhibitor CPT-11, Gemcitabine and Doxorubicin treatment [15,30]. Moreover, UCP-2 inhibitors could sensitize drug resistant leukemia cells to chemotherapeutics[31], suggesting that targeting this oncogenic protein may be a potential strategy for the treatment of chemo-resistance. In the present study, we observed that increased expression of UCP-2 in a Doxorubicin-resistant breast cancer cell line MCF-7/Dox at both mRNA and protein level compared to its parental cell line. More interestingly, this up-regulation is concomitant with the reduced expression of miR-133a, which is evidenced as a direct repressor of *UCP-2* during myogenesis and activation of inflammation [22,23]. Next, our observations implicates that exogenous expression of miR133a in Doxorubicin-resistant breast cancer cells MCF-7/Dox could confer its Doxorubicin sensitivity both *in vitro* and *in vivo*. This was accompanied with the decreased expression of UCP-2 in both mRNA and protein levels, indicating that the miR133a/UCP-2 is linked with chemotherapy-resistance in breast cancer.Furthermore, the essential role of UCP-2 in miR133a-induced Doxorubicin sensitivity was further validated by the finding that knockdown of UCP-2 could sensitize MCF-7/Dox cells to Doxorubicin treatment *in vitro*. These findings, consistent with previous studies[15,30], provide direct evidences that UCP-2 could be a critical inducer in the development of Doxorubicin resistance in breast cancer. To further elucidate the significance of miR-133a on the expression of UCP-2 in breast cancer, we found by luciferase assay that transduction of miR133a could reduce the transactivation of UCP-2 gene promoter in MCF-7/Dox cell line. This suggests that miR133a might play an essential role in chemotherapy development by its direct regulation UCP-2 expression in breast cancer cells.

As *in vitro* evidences indicated that miR133a and UCP-2 might be involved in Doxorubicin-resistance in breast cancer cells, their *in vivo* efficacy was further explored. Inhibition of tumor growth was enhanced by Doxorubicin combined with intratumoral delivering pre-miR133a compared to Doxorubicin alone. And this was accompanied by reduced expression of UCP-2 in tumor xenografts, indicating that the miR133a/UCP-2 axis could be a potential therapeutic target for breast cancer therapy.

## Supporting Information

S1 Fig Morphology of MCF-7 and MCF-7/Dox under the treatment of Doxorubicin. MCF-7 and MCF-7/Dox cells were seeded in 24-well plates at 5t 5well plates at 5ol group. three independenicin (0 and 0.8 nmol/L) and incubated for another 24 h. Obvious morphological and cell viability changes were observed after the treatment of doxorubicin of 0.8 nmol/L.
(7Z) Click here for additional data file.
